# Turning off the empathy switch: Lower empathic concern for the victim leads to utilitarian choices of action

**DOI:** 10.1371/journal.pone.0203826

**Published:** 2018-09-13

**Authors:** Reina Takamatsu

**Affiliations:** Department of Psychology and Human Developmental Sciences, Nagoya University, Aichi, Japan; University of Vienna, AUSTRIA

## Abstract

Empathy enables people to vicariously experience the other’s pain. At the same time, the focus of empathy can be narrow and reserved for a limited number of people. In sacrificial dilemmas, non-empathic people are more likely to sacrifice one person for greater good. However, no study has investigated the role of diminished empathic concern for the victim in utilitarian choices of action. In two studies, we investigated how empathy actually experienced in sacrificial dilemmas affects a decision to perform a harmful action onto the victim. In Study 1 (*N* = 275), participants were asked to rate the extent to which they were feeling two divergent tropes of affective empathy: other-oriented empathy (empathic concern) and self-oriented empathy (personal distress). Results showed that lower levels of other-focused empathy for the victim predicted utilitarian choices of action. In Study 2 (*N* = 170), participants were asked to rate the extent to which they empathized with the victim and the saved. We also assessed dispositional empathy and psychopathy to test a hypothesis that psychopathy mediates the relationship between lower empathy for the victim and utilitarian choices of action. Results supported this hypothesis, whereas dispositional empathy was not significantly correlated with utilitarian choices of action. Overall, lower empathy experienced in the dilemma situation was associated with utilitarian choices of action, and this was specific to reduced empathic concern for the victim. People choose to pursue the utilitarian end that accompanies harm onto the other as a mean when the victim is out of their empathic focus.

## Introduction

Would you sacrifice an innocent person if it saves a greater number of people? This is a question in moral dilemmas, where harm on a small number of people is a tradeoff for saving a greater number of people. A utilitarian would argue that the best choice of action produces the greatest happiness [1]. Hence, it is morally appropriate to sacrifice one individual for several others. Many would agree this contention as long as the cost of maximizing goodness is in the permissible range. In the standard trolley dilemma, the majority chooses to hit a switch on a dashboard that will cause a death of one person in order to avoid the deaths of five people [2]. In stark contrast, most people hesitate to perform the utilitarian action that involves direct harm onto an identifiable victim in sacrificial dilemmas, such as pushing a man off a bridge [3]. The latter situation is intertwined with performing a harmful action directly onto someone who is physically close. The discrepancy in the proportion of endorsing utilitarian judgment suggests that people are generally averse to harming others, and this harm aversion plays a key role in avoiding a utilitarian choice of action in moral dilemmas [4].

The dual process theory of moral judgment [5–7] posits that people arrive at utilitarian or non-utilitarian judgments by two independent processes: intuitive (i.e., affective) and cognitive. The core assumption of this theory is that non-utilitarian judgment in sacrificial dilemmas is driven by automatic affective responses, whereas deliberate cognitive processes lead to utilitarian judgment. Past studies provide support for the dual process theory. Earlier evidence comes from brain-imaging studies. Solving sacrificial moral dilemmas activated brain regions involved in emotion and social cognition [6,7]. Subsequent studies have provided support for the notion that blunted emotion leads to endorsing the utilitarian choice of action in sacrificial dilemmas. Damage to the part of the brain that causes emotional deficits alters the judgment pattern, such that patients with damage to the ventromedial prefrontal cortex are disproportionally likely to endorse utilitarian judgment in sacrificial dilemmas [8] even by sacrificing own child [9].

Emotion is a strong motivation for avoiding harm onto the other, and negative emotions and low levels of affective empathy have been found to predict utilitarian judgment [4, 6, 7] When people are less averse to harming others (i.e., under the influence of alcohol), the utilitarian deed is an easy option. Similarly, those who lack affective empathy (i.e., empathic concern) remain emotionally distant and are increasingly likely to endorse harm in sacrificial dilemmas [10–12]. The affective empathic deficit is prominent in individuals with high psychopathy traits, and low levels of empathic concern mediate the link between psychopathy and utilitarian judgment in sacrificial dilemmas [10–13]. Empathy helps one to see the emotional significance of a situation. Without empathy, those with high psychopathy take no emotional cues from a stressful situation [14] and would perceive no moral conflict in sacrificial dilemmas [15].

Empathy is a multi-faceted construct [16, 17] and susceptible to interpersonal cues in a given situation [18–19]. Past studies have shown that utilitarian judgment in sacrificial dilemmas is well predicted by empathic concern, which is one type of affective empathy [10, 13, 20]. However, the relationship between trait empathy and utilitarian bias has not been consistently found [20, 21]. To be more specific, state empathy is not a robust predictor of utilitarian judgment although empathy studies have been using state empathy as the index of the extent to which an individual actually experience empathy in response to unfortunate others [21]. Accumulating evidence shows that dispositional empathy might not be reliable for predicting an individual’s behavior because the extent to which people feel empathy for others largely depends on the type of empathy and situation [18, 20, 21].

Empathy comes in many variations, and empathy studies have used different dimensions, including but not limited to state/trait, other-focused/self-focused, and affective/cognitive, to better capture its multi-dimensionality [16, 17]. For instance, affective empathy has two forms: other focused, empathic concern and self-focused, personal distress, and they have divergent roles in shaping one’s behavior [17]. In sacrificial dilemmas, trait empathic concern is associated with non-utilitarian judgment, but the latter is not [10, 13]. Studies investigating the relationship between state emotion and utilitarian bias in sacrificial dilemmas have not taken into account the type of state empathy on this dimension. In Study 1, we endeavored to extend on past findings by measuring other-focused and self-focused empathy as state constructs. We predicted that other-focused empathy is associated with non-utilitarian judgment in sacrificial dilemmas, whereas self-focused empathy is not.

To fully understand the effect of empathy on decision-making processes, we should consider not only the type of empathy, but also the focus of empathic concern. People tend to empathize with a familiar or likable individual, while failing to recognize the need of distant or disliked others [19, 22–24]. In sacrificial dilemmas, there are several people in the life-or-death situation: a victim who would be sacrificed for the greater good (the victim) and several others who would be saved by the utilitarian choice of action (the saved). Past findings on partial nature of empathy suggest that on many occasions, people narrow down empathic concern to one or the other [22, 24–26]. In sacrificial dilemmas, people would empathize with either the victim or the saved before making a choice. One study investigating the relationship between attachment styles and utilitarian judgment in sacrificial dilemmas has shown that an individual who lacks empathy for the identifiable victim or shows greater empathy for the saved endorses harm in sacrificial dilemmas [23]. Thus, the focus of empathy can be either the victim or the saved in sacrificial dilemmas. In Study 2, we predicted that reduced empathy for the victim in sacrificial dilemmas leads to utilitarian choices of action. We also predicted that higher empathy for the saved would be linked to utilitarian choices.

For Study 1 and 2, we conducted online surveys. Ethical approval for both studies was granted by the Nagoya University Research Ethics Committee.

## Study 1: Effects of other- and self-focused empathy for the victim on utilitarian choice

Past studies have shown that there are 2 divergent tropes of affective empathy: empathic concern and personal distress [16]. Empathic concern is an other-oriented empathy that predicts a pure altruistic motivation for helping. In contrast, personal distress is a self-absorbed empathy that reduces the likelihood of helping when a ratio of the cost is greater than the benefit. Past findings have shown that in sacrificial dilemmas, empathic concern, but not personal distress, predicts utilitarian judgment [10, 13]. In Study 1, we investigated the role of affective empathy elicited in response to reading sacrificial dilemmas. We predicted that empathic concern, experienced in the dilemma situation affects utilitarian choices of action.

## Methods

All procedures performed in studies involving human participants were in accordance with the ethical standards of the institutional and/or national research committee and with the 1964 Helsinki declaration and its later amendments or comparable ethical standards. Informed consent was obtained from all individual participants included in the study. After providing informed consent, participants completed an online questionnaire.

### Participants

Participants were 477 Amazon’s Mechanical Turk workers paid 50 cents. Respondents who could not respond to the control question properly (i.e., “Choose ‘Extremely’ for this question”) and those who spent less than three minutes to complete the survey were excluded from the data analysis (% retained: 57.7%). The final sample consisted of 275 (43% female; *Mage* = 37.70, *SD* = 11.95). They were varied in ethnicity (53% White, 19% Asian, 14% Hispanic/Latino, 5% Black, and 7% other or mixed ethnic heritage).

### Measures

#### Utilitarian choices of action

Participant read 2 dilemma scenarios from a past study [6] (*footbridge* and *raftboat*) in the randomized order and responded to whether they would carry out the action in question to save more people on a 4-point scale from 0 (*definitely no*) to 3 (*definitely yes*).

#### Other-focused and self-focused empathy

After making a choice, all participants were asked to indicate the extent to which they were feeling each of 10 emotions toward the victim on a 7-point scale from 1 (*not at all*) to 7 (*extremely*). Five of 10 emotions were adjectives that formed an empathic concern index (*sympathetic*, *compassionate*, *concerned*, *empathic*, *and tender*). The rest were five adjectives that formed a self-oriented sadness index (*low-spirited*, *heavy-hearted*, *sad*, *sorrowful*, *melancholy*). These emotion measures were adapted from past studies [27, 28]. Cronbach alphas of other-focused empathy and self-focused empathy were .82 and .85 for the footbridge dilemma and .85 and .81 for the raftboat dilemma respectively.

## Results and discussion

### Preliminary analysis

The results of the Shapiro-Wilk test of normality showed that outcome variables: utilitarian choices of action in the footbridge and raftboat dilemmas were not normally distributed (i.e., the Shapiro-Wilk test was significant at *p* < .001). Therefore, we decided to treat these outcome variables as ordinal and run non-parametric test.

Table 1 shows means, standard deviations, medians, range, Cronbach alphas, and gender differences for observed variables. There were gender differences in several variables, including utilitarian choice of action in the raftboat dilemma, *Z* = − 2.86, *p* = .004; therefore, gender was entered as a covariate for later analysis. To examine whether a significant difference exists in utilitarian choices of action in the footbridge and raftboat dilemmas, a Wilcoxon signed-rank test was performed. The result showed that people were more likely to sacrifice one person in the raftboat dilemma where their own lives are saved by the utilitarian choice, *Z* = −8.29, *p* < .0001.

**Table 1:** Means, standard deviations, medians, minimum-maximum range, cronbach alphas, and gender differences for key variables.

Key variables	Mean (*SD*)	Min, max	Cronbach alpha	Gender differences (*t*, *Z*)
Footbridge dilemma				
Other-focused emotion	23.43 (6.70)	5, 35	.82	−2.58**
Self-focused emotion	21.12 (7.50)	5, 35	.85	−1.46
Utilitarian choice	1.09 (.93)	0, 3	−−	−3.99***
Raftboat dilemma	21.92 (5.79)			
Other-focused emotion	24.75 (6.86)	5, 35	.85	−5.19***
Self-focused emotion	23.81 (6.82)	5, 35	.81	−3.63***
Utilitarian choice	1.61 (.93)	0, 3	−−	−2.86**
Other-focused emotion	48.18 (12.22)	6, 30	.84	−4.32***
Self-focused emotion	44.93 (12.88)		.83	−3.27***
Utilitarian choice	2.70 (1.63)	0, 6	−−	3.95***

*Note*. N = 272. p* < .05, p** < .01, p*** < .001. *t* = two sample independent t-test. *Z* = two sample independent Mann-Whitney’s test. Gender has been dummy coded as 0 = male, 1 = female.

### Main analysis

We ran logistic regression to test associations between the ordinal outcome variable (utilitarian choices of action) and key variables (other-focused and self-focused empathy). Gender was entered as a covariate. Table 2 illustrates this result.

**Table 2:** Logistic regression results for Study 1: Other-focused and self-focused empathy as predictors of utilitarian choices of action in two sacrificial dilemmas.

Predictor variable	Dilemma type	*b* [95% CI]	Wald	*p*
Other-focused empathy	Other-beneficial	−.073 [−.0114, −.031]	11.96	.001
	Self-beneficial	.081 [.043, .118]	17.97	< .0001
Self-focused empathy	Other-beneficial	−.095 [−.136, −.053]	20.02	< .0001
	Self-beneficial	.068 [.027, .109]	10.68	.001

*Note*. N = 272. *b* = Logit coefficient, CI = Confidence Interval.

For both dilemma types, empathy experienced in the dilemma situation predicted utilitarian choices of action. In the footbridge dilemma, the likelihood of utilitarian choice increased when other-focused empathy or self-focused empathy is decreased by one unit (other-focused empathy: *b* = −.073, Wald = 11.96, *p* = .001; self-focused empathy: *b* = −.095, Wald = 20.02, *p* < .0001). In the raftboat dilemma, the likelihood of utilitarian choice increased when other-focused empathy or self-focused empathy is increased by one unit (other-focused: *b* = .081, Wald = 17.97, *p* = < .0001; self-focused: *b* = .068, Wald = 10.68, *p* = .001).

In line with previous studies [10, 13, 25], we found that other-focused empathy negatively predicted utilitarian choices of action in the other-beneficial dilemmas. However, self-focused empathy also predicted the utilitarian bias. One possible explanation is that making a utilitarian choice would release the emotional distress elicited in sacrificial dilemmas. Past studies have found that egoistic people would intervene to help others if they cannot easily retreat from the situation [16]. In Study 1, participants only needed to choose (clicking one of the choices on the screen) whether they would or would not take the action in question. Because making a utilitarian choice does not entail personal costs on the judgment task, it would have been a selfishly motivated choice of action.

An interesting finding was that other-focused and self-focused empathy negatively predicted utilitarian choices of action in the other-beneficial dilemma. In contrast, the two empathy items positively predicted utilitarian choices of action when the dilemma involved self-benefit. The present findings and others [23, 25] highlight the role of motivation that is contingent on the dilemma situation in determining one’s willingness to perform a harmful action onto another. To probe into the role of motivation, we set up the second study.

## Study 2: Empathy for specific people in sacrificial dilemmas

The results of Study 1 showed that lower levels of empathic concern and higher levels of personal distress experienced in sacrificial dilemmas predicted utilitarian choices of action. A limitation of Study 1 is that empathy for the victim was not assessed explicitly. In Study 1, the empathy items distinguished the two types of affective empathy (empathic concern, personal distress) from one another, but an individual could have empathized for the victim, the saved, or both simultaneously. To address this concern, we included items to assess levels of empathic concern for the victim and saved in sacrificial dilemmas, given that empathy is susceptible to interpersonal cues [18, 19, 22, 24, 26]. Based on the results of Study 1 that both empathic concern and personal distress affected utilitarian bias, we only measured empathic concern in Study 2.

Moreover, we sought to extend Study 1 in two ways. First, we included a measure of dispositional empathic concern, as well as empathy experienced in sacrificial dilemmas for specific targets: empathy for the victim and the saved. In this vein, we tested the prediction that lower empathy for the victim in sacrificial dilemmas would lead to utilitarian choices of action. We also predicted that empathy for the victim would positively predict utilitarian choices of action when the dilemma implicates the respondent’s own life (i.e., self-beneficial dilemma). In contrast, the empathy would predict utilitarian choices negatively when the act is aimed at promoting other people’s welfare.

We also included a self-report scale that assesses psychopathy. Past studies have shown that individuals with lower levels of empathic concern are increasingly more likely to endorse harm onto the other in sacrificial dilemmas [13]. Do people with high psychopathy endorse utilitarian choices of action because they lack empathy for others in general? Or do they only feel less empathy for the victim? Empathic deficit in psychopathy is specific. People with high psychopathy are skilled in understanding others’ mental states for exploitation while showing no concern for the victim [29, 30]. Their primary concern is maximizing personal gains rather than protecting vulnerable people [12, 31, 32]. These suggest that people with high psychopathy would be selective with whom to empathize. We predicted that lower levels of empathy for the victim would mediate the relationship between psychopathy and utilitarian choices of action.

In addition to testing the prediction, we probed into justifications for moral judgments to gain insights into psychopathy and permissibility of harm. The rational process of moral judgment is currently an understudied area although past studies have shown that rational thinking may reflect utilitarian judgment in moral dilemmas [5, 7, 33, 34]. Psychopathic people would make utilitarian judgments for non-moral motivations [12, 31, 35], and simply asking to choose between a utilitarian and non-utilitarian action in sacrificial dilemmas may reveal little or nothing about morality [36]. For these reasons, we felt that studying the rational processes in moral judgment is useful for people with high psychopathy who are not bound to moral values. Based on past studies [37–39], we chose five justification variables (deontological ethics, descriptive norm, emotional reactivity, selfish concern, and confidence) that would influence utilitarian choices of action in sacrificial dilemmas. Specifically, we were interested in how empathy is related to justification of harm in sacrificial dilemmas.

## Methods

All procedures performed in studies involving human participants were in accordance with the ethical standards of the institutional and/or national research committee and with the 1964 Helsinki declaration and its later amendments or comparable ethical standards. Informed consent was obtained from all individual participants included in the study. After providing informed consent, participants performed the online task and filled out a questionnaire.

### Participants

Participants were 208 Amazon’s Mechanical Turk workers paid 50 cents. Given the sampling issues in Study 1, participation for Study 2 was limited to respondents with a reliable record on the MTurk crowdsourcing system, called “Masters”, who had established themselves as quality respondents. Additionally, respondents who could not respond to the control question properly (i.e., Read a paragraph and choose an appropriate answer, “None of the above” from multiple choices) and those who spent less than five minutes to complete the survey were excluded from the data analysis (% retained: 81.7%). The final sample consisted of 170 (45% female; *Mage* = 37.70, *SD* = 11.95). They were varied in ethnicity (75% White, 12% Asian, 5% Hispanic/Latino, 4% Black, and 3% other or mixed ethnic heritage).

### Measures

#### Utilitarian choices of action

Six sacrificial moral dilemma scenarios from a past study [6] were selected (*Footbridge*, *Crying Baby*, *Vaccine*, *Raftboat*, *Modified Safari*, and *Sophie’s Choice*). The task consisted of three parts. First, participants read a dilemma scenario and responded to whether they would carry out the action in question to save more people (e.g., In this situation, would you push the stranger on to the tracks in order to save the five workmen?). Participants responded to the question on a 4-point scale from 0 (*definitely no*) to 3 (*definitely yes*).

#### Empathic concern for each target

Immediately after making a decision, participants rated the extent to which they empathize with each person in the dilemma situation: the saved and the victim (e.g., The five working men, The stranger in the Footbridge dilemma) on a 5-point scale from 1 (*not at all*) to 5 (*to a great extent*).

#### Justifications for utilitarian choices of action

To examine how people reason about utilitarian judgment, participants were asked to imagine that they have made a utilitarian judgment, irrespective of the initial judgment (e.g., Now, imagine that you push the stranger on to the tracks in order to save the five workmen. As a consequence, the stranger is killed by the runaway trolley. Please indicate the extent to which you use the following five types of reasoning to agree or disagree with the decision). Participants responded to five types of reasoning: 1) deontological ethics; 2) descriptive norm; 3) emotional reactivity; 4) selfish concern, and 5) confidence. There were five justification items for each dilemma, hence five items per justification in total. The question items for justification variables for the footbridge dilemma are shown in Table 3 as an example.

**Table 3:** Sample questions and responses for justification variables (footbridge dilemma).

Types of justification	Questions
Deontology	Pushing the stranger on to the tracks is immoral because this act contradicts principles one has to follow.
Moral relativity	The majority would sacrifice the stranger to save the five workmen.
Emotional reactivity	The thought of me pushing the stranger on to the tracks is overwhelming.
Egoistic concern	I do not care much about the stranger and five workmen only if I am safe and sound.
Confidence	I trust my judgment in the situation; reverse-coded.

*Note*. The instruction was “Now, imagine that you pushed the stranger on to the tracks in order to save the five workmen. As the consequence, the stranger was killed by the runaway trolley. Please indicate your feeling and attitudes toward the situation.”

The wording of justification questions was adjusted for each dilemma.

These items were based on past studies [37–39]. Responses were recorded on a 5-point scale from 1 (*strongly disagree*) to 5 (*strongly agree*) and were averaged to create a composite measure of five constructs in interest.

#### Primary psychopathy

Participants completed a subscale of Levenson Self-Report Psychopathy Scale (LSRP) [40], which measures primary psychopathy traits (emotional deficit and poor interpersonal functioning) and secondary psychopathy traits (antisocial lifestyle and behavioral patterns) in non-clinical samples. Only items for primary psychopathy were used because past studies have shown that utilitarian judgment in sacrificial dilemmas is associated with primary psychopathy, but not with secondary [11]. Responses were recorded on a 4-point scale from 0 (*disagree strongly*) to 3 (*agree strongly*).

Given that the core features of psychopathic traits fall along a continuum of severity [41], we took a dimensional approach to the study of psychopathic traits in non-clinical populations. There is a subgroup of people with high psychopathic traits in the general population, suggesting the psychopathic traits are heterogeneous [42–44]. To capture the spectrum of psychopathic traits, we treated psychopathy scores as continuous, rather than a diagnostic category.

#### Empathic concern

Participants completed a subscale of Interpersonal Reactivity Index (IRI) [17], which assesses an individual’s general tendency to feel sympathy and concern for unfortunate others, namely dispositional affective empathy. Past studies have shown that the affective component of empathy best predicts the tendency to avoid direct harm onto the other in dilemma situations [10, 13]. Responses were recorded on a 5-point scale from 1 (*not at all characteristic of me*) to 5 (*extremely characteristic of me*).

## Results and discussion

Table 4 shows means, standard deviations, medians, range, Cronbach alphas, and gender differences for observed variables.

**Table 4:** Means, standard deviations, medians, minimum-maximum range, cronbach alphas, and gender differences for key variables.

Key variables	Mean (*SD*)	Min, max	Cronbach alpha	Gender differences (*t*)
Primary psychopathy	15.99 (8.45)	1, 42	.89	5.40***
Empathic concern	26.29 (6.46)	7, 35	.89	−5.17***
Empathy for the saved	25.87 (4.77)	6, 30	.89	−2.52*
Empathy for the victim	25.19 (5.02)	6, 30	.89	−3.78***
Deontology	21.92 (5.79)	6, 30	.88	−2.34*
Moral relativity	20.17 (4.69)	6, 30	.81	.26
Emotional reactivity	24.66 (5.69)	6, 30	.92	−3.88***
Egoistic concern	10.48 (4.87)	6, 26	.90	2.13*
Confidence	21.25 (5.31)	6, 30	.89	2.08*
Utilitarian choice	14.89 (4.04)	6, 24	.84	2.30*

*Note*. N = 170. p* < .05, p** < .01, p*** < .001. *t* = two sample independent t-test. Gender has been dummy coded as 0 = male, 1 = female.

Table 5 shows correlations among observed variables. As predicted, reduced empathy for the victim was significantly associated with utilitarian choices (empathy for the victim: *r* = −.22, *p* = .004), but empathy for the saved was not, *r* = −.083, *p* = .29. Dispositional empathy was not significantly associated with utilitarian choices, *r* = −.14, *p* = .069. Empathy for the victim was significantly associated with the tendency to refute utilitarian choices on the basis of deontology and emotional reactivity (empathy for the victim: *r* = .41, *p* < .0001; emotional reactivity: *r* = .63, *p* < .0001). In contrast, reduced empathy for the victim was significantly associated with egoistic concern (*r* = .60, *p* < .0001).

**Table 5:** Intercorrelations among all variables in Study 2.

Variables	1	2	3	4	5	6	7	8	9	10
1. LSRP-1	−	−.64***	−.51***	−.54***	−.14^†^	−.009	−.52***	.53***	.23**	.18*
2. EC	−	−−	.49***	.52***	.18*	−.024	.40***	−.51***	−.098	−.14^†^
Empathy for:	−	−−	−−	−−						
3. The saved	−	−−	−−	.86***	.35***	.16*	.58***	−.52***	.048	−.083
4. The victim	−	−−	−−	−−	.41***	.091	.63***	−.60***	.023	−.22**
Justifications:	−	−−	−−	−−	−−					
5. Deontology	−	−−	−−	−−	−−	−.19*	.47***	−.17*	.021	−.57***
6. Relativity	−	−−	−−	−−	−−	−−	.033	−.061	.16*	.47***
7. Reactivity	−	−−	−−	−−	−−	−−	−−	−.39***	−.11	−.26**
8. Egoistic	−	−−	−−	−−	−−	−−	−−	−−	.93	.11
9. Confidence	−	−−	−−	−−	−−	−−	−−	−−	−−	−.011
10. U. choice	−	−−	−−	−−	−−	−−	−−	−−	−−	−−

*Note*. N = 170. *p*^†^ < .1, p* < .05, p** < .01, p*** < .001

LSRP-1 = primary psychopathy, EC = empathic concern, Relativity = moral relativity, Reactivity = emotional reactivity, Egoistic = egoistic concern, U. choice = utilitarian choice.

Psychopathy was significantly associated with lower levels of empathy experienced in sacrificial dilemmas (empathy for the victim: *r* = −.54, *p* < .0001; empathy for the saved: *r* = −.51, *p* < .0001). Psychopathy was also significantly associated with lower levels of dispositional empathy, *r* = −.64, *p* < .0001. There were gender differences in several variables, including utilitarian choices of action, *t* = 3.95, *p* < .001; therefore, gender was entered as a covariate for later analysis.

In a follow-up study, we administered a short version of the questionnaire used in Study 2 to a student sample. We again found that lower empathy for the victim and higher empathy for the saved predict utilitarian preferences in the sacrificial dilemma (See S1 File and S1 Table for details).

### Main analysis

#### Empathy and utilitarian choices of action

We ran logistic regressions to test associations between utilitarian choices of action in self- and other-beneficial dilemmas and empathy for the victim with gender as a covariate. To replicate Study 1, utilitarian choices in the footbridge and raftboat dilemmas (other-beneficial and self-beneficial respectively) were used for this analysis because dilemma scenarios vary in content [34]. The results showed that empathy for the victim negatively predicted utilitarian choices of action in both types of dilemmas (other-beneficial: *b* = −.19, Wald = 11.00, *p* = .001; self-beneficial: *b* = −.13, Wald = 4.89, *p* = .027). In comparison, empathy for the saved positively predicted utilitarian choices in both dilemmas (other-beneficial: *b* = .14, Wald = 5.47, *p* = .019; self-beneficial: *b* = .12, Wald = 4.07, *p* = .044). Unlike Study 1, effects of empathy on utilitarian choices were dependent on whom to feel empathic concern for rather than the dilemma type.

#### Empathy and post-hoc justifications

To ascertain what kinds of justifications are associated with empathy for the victim, regression analysis was conducted. Deontology and emotional reactivity positively predicted empathy for the victim (deontology: B = .14, *p* = .006; emotional reactivity: B = .33, *p* < .0001). In comparison, egoistic concern negatively predicted the empathy (B = −.42, *p* < .0001). Moral relativity and confidence were not associated (moral relativity: B = .082, *p* = .15; confidence: B = .062, *p* = .22). Table 6 depicts the results.

**Table 6:** Regression results for Study 2: Justification variables as predictors of empathy for the saved and empathy for the victim.

	Outcome variables		
	Empathy for the victim		
Predictor variables	B	*SE*	95% CI
Deontology	.15**	.052	[.042, .247]
Moral relativity	.082	.057	[−.031, .195]
Emotional reactivity	.33***	.056	[.219, .441]
Egoistic concern	−.42***	.057	[−.529, −.303]
Confidence	.062	.050	[−.037, .160]
*R*^*2*^	.76		

*Note*. N = 170. p* < .05, p** < .01, p*** < .001

B = unstandardized coefficient, CI = Confidence Interval.

#### Mediation analysis

We conducted a mediation analysis to test the hypotheses. The 95% bias-corrected bootstrapping mediation test was conducted using the INDIRECT macro for SPSS with 5000 re-samplings [45, 46]. The mediation effect is significant if the confidence interval does not include zero. Based on results of the preliminary analysis, gender was entered as a covariate. We entered empathy for the victim, empathy for the saved, and empathic concern as mediating variables.

**Fig 1:**
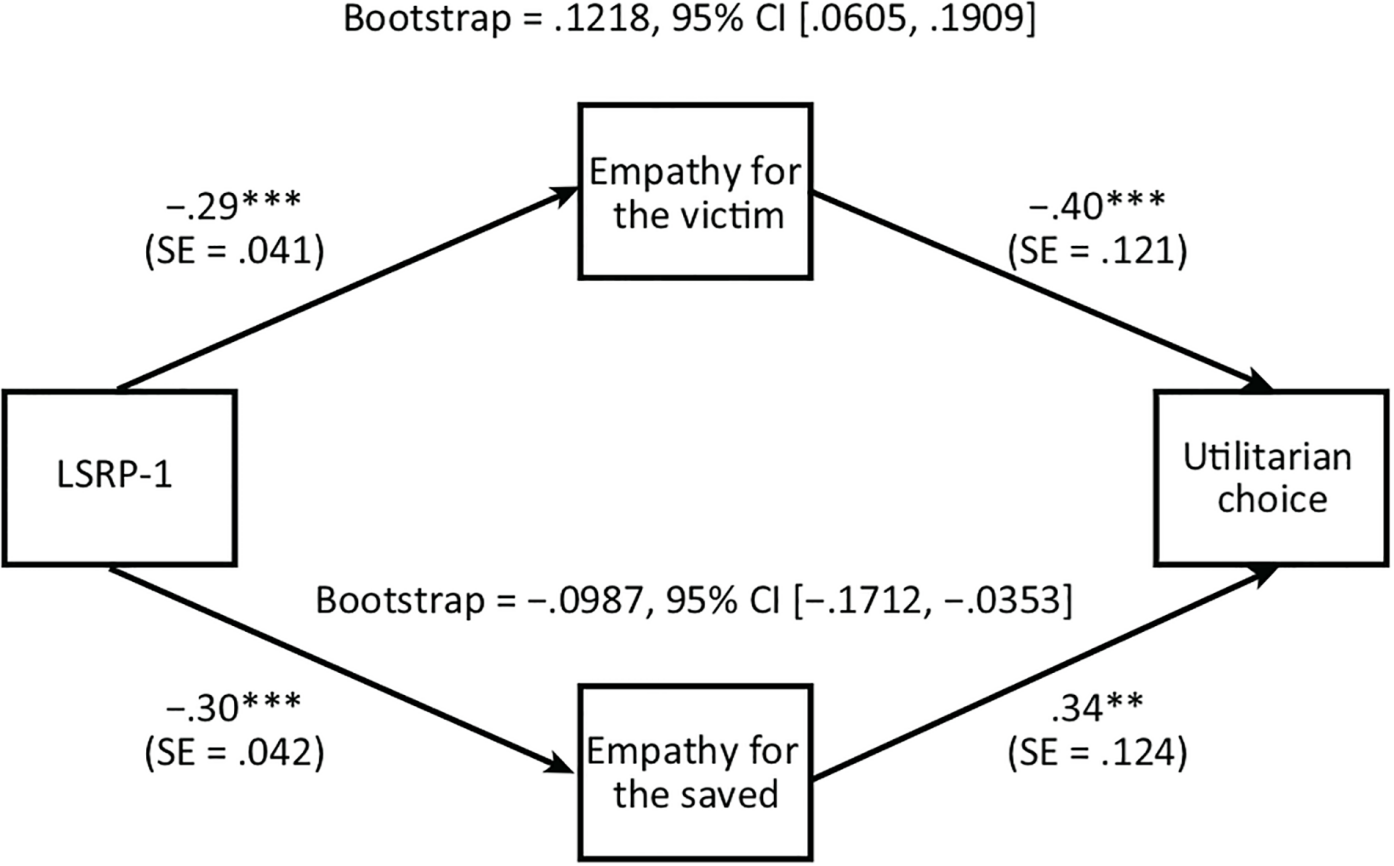
Direct and indirect effects predicting utilitarian choice, empathy for two targets (the victim, the saved) as predictors. p* < .05, p** < .01, p*** < .001. CI = confidence interval.

Results showed that the relationship between psychopathy and utilitarian choices of action was fully mediated by empathy for the victim and empathy for the saved (empathy for the victim: standardized effect size = .1218, 95% CI [.0605, .1909]; empathy for the saved: standardized effect size = −.0987, 95% CI [−.1712, −.0353]). The direct effect of psychopathy was no longer significant after mediating variables were included in the model (*p* = .58). Empathy concern was not significant as a mediator (95% CI [−.0610, .0485]). Fig 1 illustrates the results.

#### Post-hoc justifications for utilitarian judgment

We tested correlations between psychopathy and justification variables. Psychopathy was significantly associated with three of the justification variables: emotional reactivity, self-interest, and confidence (emotional reactivity: *r* = −.52, *p* < .0001; self-interest: *r* = −.53, *p* < .0001; confidence = *r* = −.23, *p* < .01).

Next, we ran another mediation analysis to test whether emotional reactivity mediates the relationship between psychopathy and utilitarian judgment. Among the five justification variables, only emotional reactivity was correlated with both variables in interest. The results showed that the relationship between psychopathy and utilitarian judgment was fully mediated by emotional reactivity (standardized effect size = .0484, 95% CI [.0102, .0967]). After the mediating variable was entered in the model, the direct effect of psychopathy on utilitarian judgment was no longer significant (*p* = .72).

## General discussion

In two studies, we sought to explain how empathy influences utilitarian choices of action in sacrificial dilemmas. Under the assumption that empathy is susceptible to affective cues in the context, we measured empathy experienced in dilemma situations (i.e., state empathy) to better understand how empathy for the victim affects one’s decision to perform a harmful act for the utilitarian end. Overall, we found that lower empathic concern for the victim predicted utilitarian choices of action.

In Study 1, we asked participants to report the extent to which they are feeling ten emotions that represent empathic concern or personal distress. Results of Study 1 showed that lower other- and self-focused empathy scores (empathic concern, personal distress) were associated with utilitarian choices of action when the harmful act was aimed at saving the lives of several others, including the respondent’s own life. In contrast, higher empathy scores were associated with utilitarian choices when the dilemma involved no self-interest. In line with past findings that empathy is susceptible to the context [18, 19, 26], overall results suggest that the effect of state empathy depends on the situation rather than whether empathy is self or other-oriented.

In Study 2, we included two measures that assessed dispositional empathy and empathy for specific individual(s) to better understand how empathy experienced temporarily in sacrificial dilemmas affects an individual’s decision to carry out the harm. In doing so, we also investigated the relationship between psychopathic traits and utilitarian choices of action. Psychopathy is a robust predictor of utilitarian choices in sacrificial dilemmas, and we were interested in testing the relationship by including empathy for the victim and saved as mediators. Results of Study 2 showed that empathy for the victim, but not empathy for the saved, was associated with utilitarian choices of action. Specifically, an individual who lacks empathic concern for others makes utilitarian choices because of lower empathy for the victim and higher empathy for the saved.

In Study 2, we also investigated what types of justifications for utilitarian choices influence empathy and found that deontology and emotional reactivity were associated with empathy for the victim. The significant relationship between emotional reactivity and empathy gives support to the notion that empathic reaction plays a key role in utilitarian choices in sacrificial dilemmas [20, 21]. As to deontology, the preexistent belief in the duty-based ethics diverts one’s attention away from the utilitarian solution [38]. The non-utilitarian choice is quick and affect-laden, and one must question the initial conclusion to weigh pros of non-utilitarian solution against the cons [6, 7]. However, adapting the deontological approach might narrow down the individual’s scope of empathic concern. In sacrificial dilemmas, one’s judgment and choice are influenced not only by state emotion but also by thinking style, religious belief, and identification with others [23, 39, 47]. The present findings suggest that empathy for the victim is influenced by a wide array of intrapersonal and contextual factors. Having a deontological perspective discourages the respondent from thinking twice by increasing empathy for the victim, which in turn leads to non-utilitarian choice.

For those who lack empathy for the victim, utilitarian choices of action may only reflect low action aversion, impulsivity, or lack of insight into the effect of their inconsiderate behavior on others [12, 31, 35]. Hence, the conventional approach to the study of moral judgment in sacrificial dilemmas might not be sufficient to uncover the motivation behind their utilitarian preference that may be foreign to the normative ethics. For this reason, we probed into justifications for utilitarian choice to better understand what motivates people with high psychopathy to choose the utilitarian mean. Results showed that lower emotional reactivity, self-focused reasoning, and inflated confidence predict utilitarian choices of action among those who are high in psychopathy. These reasoning styles are consistent with theoretical conceptions of psychopathy [42, 48, 49]. In many situations, emotion acts as an indication of right and wrong, thus functioning as a moral guide [8, 50]. For people with high psychopathy, making a utilitarian choice in sacrificial dilemmas is relatively easy because it is only about choosing a larger number of saved people without taking into account the consequence of own harmful action.

Nonetheless, this method of studying decision-making in sacrificial dilemmas has some limitations as well as strengths discussed. Methodological concerns pertain to lack of insight and pathological lying among those with high psychopathic traits [48]. Especially for those who have high psychopathy traits, the post-hoc reasoning would not reflect their actual reasoning because they have low motivation for reporting precisely. Without clarifying a decision-making process in which those with high psychopathy traits arrive at utilitarian judgment, it would be difficult to identify if the dual process model of moral judgment also accounts for their judgment patterns. Although they might be reliable respondents under a condition in which a diagnosis of psychopathy has no consequences of any kind [51], future studies should use other methods or indices to clarify how psychopathic traits affect the way those people endorse harm in sacrificial dilemmas for non-moral reasons. One novel method to provide detailed accounts of decision-making processes in sacrificial dilemmas would be using dilemma scenarios that pit one of the ethical principles against self-relevant concern, such as increasing personal gains.

Furthermore, it would be better to combine a self-report measure of empathy for objective measures, such as heart rate, in order to investigate effects of reduced empathy on the perceived permissibility of harm in sacrificial dilemmas among individuals with high psychopathy. In this study, one of the novel findings was that reduced empathy toward the victim predicted utilitarian judgment rather than an individual’s ability to empathize with other people. However, as shown in Study 1 and 2, state empathy might depend on the type of dilemma or focus of empathic concern (e.g., empathy for the victim or the saved). Moreover, those with high psychopathy traits would use impression management strategies to appear socially desirable and are known as unreliable survey respondents [52]. To corroborate these findings, future studies should use both self-report and objective measures of empathy.

To corroborate these findings, future studies should experimentally manipulate the level of empathy for the victim. Past studies have shown that an instruction for perspective taking (i.e., try to imagine the target’s feeling and thinking) increases not only cognitive empathy (perspective-taking), but also affective empathy (empathic concern) for the target [53]. The manipulation of empathy is also effective for those who are generally disinterested in the other’s welfare [54]. Based on results of the present study, we expect that manipulating empathy for the victim in sacrificial dilemmas would lead to non-utilitarian choices of action. However, one obstacle for this study design would be that the instruction for perspective-taking might not increase empathy for the victim because people have a natural tendency to feel empathy for unfortunate others [28]. Generally, a large proportion of respondents make a non-utilitarian choice of action in sacrificial dilemmas [6], and it would be more interesting to elucidate situational factors that reduce empathy for the victim and lead to endorsing harm for the utilitarian end. In doing so, we will have a clearer understanding of the relationship between empathy and harm aversion that seems to be entrenched in human nature.

## Supporting information

S1 File Supporting information for the follow-up study. (DOCX)

S1 Table Logistic regression results for a follow-up study: Empathy for the victim, empathy for the saved, and dispositional empathy as predictors of utilitarian choices of action in the footbridge dilemma. (DOCX)
