# Supplementary material for: Turning off the empathy switch: Lower empathic concern for the victim leads to utilitarian choices of action
Source: PLoS One. 2018 Sep 13;13(9):e0203826. doi: 10.1371/journal.pone.0203826 (PMC6136766; doi:10.1371/journal.pone.0203826)
Supplement: S1 File — (DOCX) [file pone.0203826.s001.docx]

S1 File

Supporting Information for the Follow-up Study

The purpose of the follow-up study was to replicate one of the main findings of Study 2. The main finding was that empathy for specific individual(s) predicted utilitarian choices of action, whereas dispositional empathy did not. In the follow-up study, the sample consisted of undergraduate students (female 95.8%) who participated in exchange of extra credit. Informed consent was obtained from all individual participants included in this study. The questionnaire was administered in a paper-and-pencil format.

In this shorter version, participants read the footbridge dilemma and then were asked whether they would perform the harmful action to save more people (0: *definitely no* to 3: *definitely yes*). After making a choice, they reported the extent to which they feel empathy for: the saved and the victim (1: *not at all* to 5: *to a great extent*).After the sacrificial dilemma task, participants completed a subscale of IRI (empathic concern), which assess one’s trait empathy.

Before carrying out the main analysis, we checked Cronbach alphas. Cronbach alpha of dispositional empathy (7 items) was low (α = .55). Based on results of factor analysis, we excluded one item (“I would describe myself as a pretty soft-hearted person”), and it increased the alpha to .60. We used the six items for analysis. We conducted additional analysis and found that exclusion of the item did not significantly change the main finding.

S1 Table shows the results of logistic regression with empathy items (empathy for the victim/saved, dispositional empathy) predicting utilitarian choices of action. In this sample, empathy for the victim and saved predicted utilitarian choices of action better than dispositional empathy.
