# Supplementary material for: Turning off the empathy switch: Lower empathic concern for the victim leads to utilitarian choices of action
Source: PLoS One. 2018 Sep 13;13(9):e0203826. doi: 10.1371/journal.pone.0203826 (PMC6136766; doi:10.1371/journal.pone.0203826)
Supplement: S1 Table — (DOCX) [file pone.0203826.s002.docx]

**S1 Table. Logistic Regression Results for a Follow-up Study: Empathy for the Victim, Empathy for the Saved, and Dispositional Empathy as Predictors of Utilitarian Choices of Action in the Footbridge Dilemma.**

| Predictor variable | *b* [95% CI] | Wald | *p* |
| --- | --- | --- | --- |
| Empathy for the victim | −.51 [−.882, −.135] | 7.12 | .008 |
| Empathy for the saved | .82 [.414, 1.235] | 15.48 | <.0001 |
| Dispositional empathy | −.17 [−.334, −.015] | 4.59 | .032 |

*Note*. N = 72. *b* = Logit coefficient, CI = Confidence Interval.
